# Amentoflavone Attenuates *Clostridium perfringens* Gas Gangrene by Targeting Alpha-Toxin and Perfringolysin O

**DOI:** 10.3389/fphar.2020.00179

**Published:** 2020-02-28

**Authors:** Shui Liu, Xiaofeng Yang, Hong Zhang, Jian Zhang, Yonglin Zhou, Tingting Wang, Naiyu Hu, Xuming Deng, Xiaoxue Bai, Jianfeng Wang

**Affiliations:** ^1^ Cadre’s Ward, The First Hospital of Jilin University, Jilin University, Changchun, China; ^2^ Key Laboratory of Zoonosis Research, Ministry of Education, College of Veterinary Medicine, Institute of Zoonosis, Jilin University, Changchun, China; ^3^ Department of Pathogenic Biology and Immunology, School of Basic Medical Sciences, Xi'an Jiaotong University Health Science Center, Xi’an, China; ^4^ College of Animal Sciences, Jilin University, Changchun, China

**Keywords:** *Clostridium perfringens*, gas gangrene, alpha-toxin, perfringolysin O, amentoflavone

## Abstract

*Clostridium perfringens* (*C. perfringens*) type A strains are the main cause of gas gangrene in humans and animals. Treatment of this lethal disease is limited, and the prognosis is not good. Alpha-toxin (CPA) and perfringolysin O (PFO) secreted by *C. perfringens* play irreplaceable roles in cytotoxicity to host cells, persistence in host tissues, and lethality of gas gangrene pathology. This work determined the influence of amentoflavone, a biflavonoid isolated from *Selaginella tamariscina* and other plants, on hemolysis and cytotoxicity mediated by CPA and PFO and evaluated the *in vivo* therapeutic effect on gas gangrene. Our data showed that amentoflavone could block the hemolysis and cytotoxicity induced by CPA and PFO *in vitro*, thereby mediating significant protection against mortality of infected mice in a mouse gas gangrene model, efficient bacterial clearance in tissues and alleviation of histological damage *in vivo*. Based on the above results, amentoflavone may be a potential candidate against *C. perfringens* infection by reducing CPA and PFO-mediated virulence.

## Introduction

*Clostridium perfringens* (*C. perfringens*) is a gram-positive, spore-forming, anaerobic bacterium that causes diverse diseases in both humans and animals, including gas gangrene (clostridial myonecrosis), gastroenteritis, and necrotic enteritis (Kiu and Hall, 2018). Clinically, *C. perfringens* is the most commonly identified cause of gas gangrene, a fatal disease caused by bacterial contamination in wounds (Stevens, 2000; Bryant and Stevens, 2010). Once the bacteria grow in tissues, the disease is followed by a very rapid spread. In war periods, many soldiers have died due to this disease (Alam and Dwivedi, 2016). In addition, the incidence of gas gangrene is high during natural disasters, such as earthquakes (Chen et al., 2011; Stevens et al., 2012). Patients infected with gas gangrene from traumatic wounds or surgery treatment in disasters have a very high mortality rate (as high as 50–80%) (Wang et al., 2013). Early recognition and aggressive treatment to establish a golden period of treatment are critical to save patients (Yang et al., 2015). The current therapeutic method is limited to antibiotic treatment. However, even with timely treatment with appropriate antibiotics, the healthy tissues near the infected site will also be destroyed (Bryant and Stevens, 2010; Hifumi et al., 2018). In some emergency cases, amputation will be utilized as a life-saving procedure (Stevens et al., 2004). Due to serious harm and limited treatment, new treatment approaches or therapeutic agents are needed.

*C. perfringens* has the ability to secrete multiple toxins and enzymes, causing pathophysiology. Among them, two main toxins, alpha-toxin (CPA) and perfringolysin O (PFO), are thought to be responsible for gas gangrene pathology (Awad et al., 1995; Stevens et al., 1997). CPA (also known as phospholipase C), encoded by the *cpa* gene, is a typing toxin produced by all strains of *C. perfringens*, which can hydrolyze cell membrane phospholipids and eventually lead to cell death and immune-mediated pathology at infected sites (Navarro et al., 2018). CPA induces constriction of blood vessels, decreasing the blood supply to host tissues and producing an anaerobic environment that fosters the growth of *C. perfringens* (Titball et al., 1999). Studies have indicated that CPA plays an essential role in gas gangrene through the use of *cpa* mutants, which display demonstrably reduced virulence in a mouse model (Awad et al., 1995). PFO, encoded by the *pfoA* gene, is identified as a lethal pore-forming cholesterol-dependent cytolysin (CDC) produced by nearly all *C. perfringens* strains (Verherstraeten et al., 2015). PFO can bind to cholesterol-containing membranes and oligomerize into a pore complex to lyse cells (Stevens and Bryant, 2002). Earlier findings suggested that PFO is important in gas gangrene pathology and that a *pfoA* mutant is unable to produce the histopathological features typical of gas gangrene (Awad et al., 2001). Furthermore, *C. perfringens* mutant strains lacking the *cpa* and *pfoA* genes did not cause gas gangrene (Awad et al., 1995), and both CPA and PFO were necessary for escape from phagosomes of macrophages and for survival of bacteria in host tissue (O’Brien and Melville, 2004). Therefore, CPA and PFO appear to show synergistic effects (Verherstraeten et al., 2013; Goossens et al., 2017) and are essential for gas gangrene (Awad et al., 1995).

**Figure 1:**
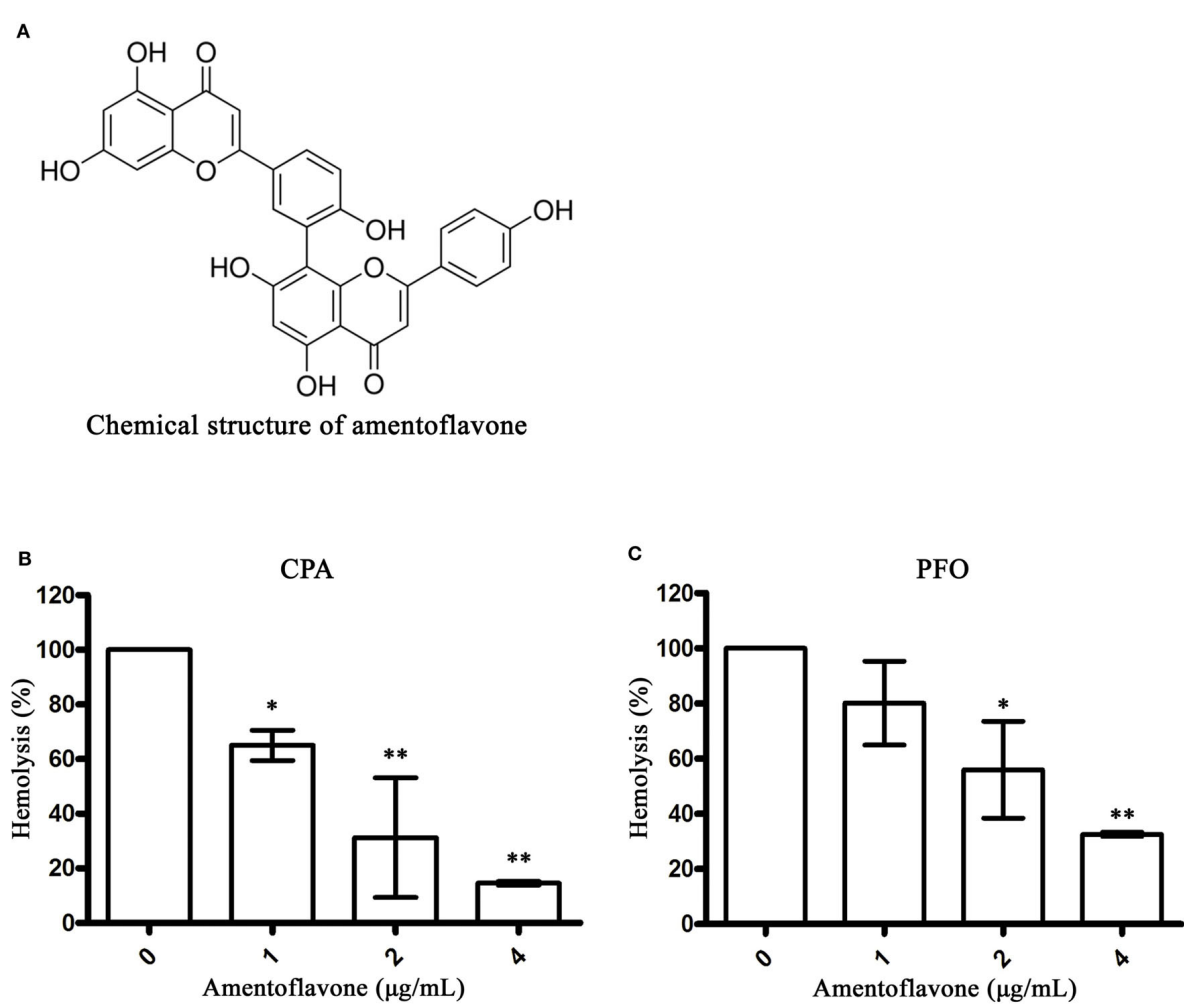
Amentoflavone inhibits hemolysis mediated by alpha-toxin (CPA) and perfringolysin O (PFO). **(A)** Chemical structure of amentoflavone. **(B)** Inhibition of CPA-induced hemolysis (%) by amentoflavone. Hemolysis assays were performed with CPA using sheep red blood cells in phosphate-buffered saline (PBS) (pH = 7.4). **(C)** Inhibition of PFO-induced hemolysis (%) by amentoflavone. Hemolysis assays were performed with PFO using rabbit red blood cells in PBS. Data are shown as the mean ± SD from three independent experiments. * indicates P < 0.05 and ** indicates P < 0.01 compared the optical density (OD) values of this group to the drug-free group.

Amentoflavone (**Figure 1A**) is a common biflavonoid isolated from a variety of traditional Chinese medicines, such as *Selaginella tamariscina* or *Ginkgo biloba* (Yu et al., 2017). Previous studies have indicated that amentoflavone has multiple pharmacological effects, including anti-inflammatory (Zhang et al., 2015), antioxidant (Saroni Arwa et al., 2015), antivirus (Li et al., 2019), and antitumor activities (Lee et al., 2018). However, to our knowledge, the effect of amentoflavone on *C. perfringens* toxins has not been reported. Because CPA and PFO are two important toxins involved in gas gangrene (Awad et al., 2001), this study was designed to assess the inhibitory effects of amentoflavone on CPA and PFO activities, to explore the ability of amentoflavone to prevent cell injury induced by these two toxins, and to examine the therapeutic protection of amentoflavone in an experimental mouse model of gas gangrene.

## Materials and Methods

### Bacterial Strain and Chemicals

*C. perfringens* ATCC13124 (control, type A strain) was commercially obtained from the American Type Culture Collection (ATCC). Amentoflavone (≥98% pure) was purchased from Chengdu Herbpurify Co., Ltd. (Chengdu, China) and dissolved in dimethyl sulfoxide (DMSO) (Sigma Aldrich, St. Louis, MO, USA). CPA protein was purchased from Sigma Aldrich.

### Protein Purification

*E. coli* BL21(DE3) carrying a pET-28a-PFO plasmid that encodes the intact *pfoA* gene (without a signal peptide) was stored in our laboratory. Protein expression of PFO was performed according to the methods of Rossjohn et al. (1997).

### Hemolytic Activity Assays

Hemolytic activity assays were performed according to previous study (Zhao et al., 2017). Briefly, purchased CPA or purified PFO in 975 μl of phosphate-buffered saline (PBS) buffer (pH = 7.4) was preincubated with a sequence of concentrations of amentoflavone (1, 2, or 4 μg/ml) at 37°C for 30 min in tubes. Then, 25 μl of sheep or rabbit red blood cells (Zhengzhou Kowloon Biological Products Co., Ltd., Zhengzhou Province, China) were added to each tube. After another incubation at 37°C for 10 min, all samples were centrifuged at 10,000 × g for 2 min to remove intact red cells. The absorbance of cell-free supernatants was measured at 540 nm. Simultaneously, in the group with concentration of amentoflavone at 0 μg/ml, red blood cells were incubated with toxin but without amentoflavone (served as positive control, with a hemolysis rate of 100%). The hemolysis rate of each group was calculated by comparison the absorbance of cell-free supernatants at 540 nm with the positive control supernatants.

### Cytotoxicity Assays

Human epithelial colorectal adenocarcinoma (Caco2) cells (ATCC HTB-37) were cultured in Roswell Park Memorial Institute (RPMI)-1640 medium (Invitrogen, Carlsbad, CA, USA) supplemented with 10% fetal bovine serum (Bioindustries, Israel) in a 5% CO_2_ atmosphere. Cells were seeded at a density of 2 × 10^4^ cells/well in a 96-well plate. The cytotoxicity of amentoflavone on Caco-2 cells was determined using lactate dehydrogenase (LDH) release assays. The influence of amentoflavone on CPA- or PFO-mediated cytotoxicity was determined using LDH release and LIVE/DEAD assays according to Liu et al. (2015). In the cytotoxicity of amentoflavone on Caco-2 cells assays, cells were treated with different concentrations of amentoflavone (ranging from 0 to 64 μg/ml). In amentoflavone treated groups, cells were added with purchased CPA (3 μg/well) or purified PFO (5 μg/well) and then treated with different concentrations of amentoflavone (ranging from 2 to 16 μg/ml). In positive control groups, cells were treated with 0.2% Triton X-100 instead of toxin (served as 100% LDH release) and in negative control groups, cells were treated without toxins or 0.2% Triton X-100 (served as 0% LDH release). Followed by incubation at 37°C for 6 h, the LDH release assays and the LIVE/DEAD assays were detected according the methods proposed by the manufacturer.

### Mouse Gas Gangrene Model

Female specific pathogen free BALB/c mice (6–8 weeks old, weighing approximately 20 g) were obtained from the Liaoning Changsheng Bio-technology Company (Liaoning Province, China) and maintained in accordance with the guidelines of the Jilin University Institutional Animal Care Committee.

Mice were infected intramuscularly in the thigh muscle with *C. perfringens* ATCC13124 (2 × 10^8^ bacteria for survival assays and 1 × 10^7^ bacteria for bacterial burden and histopathology determination). After infection, mice were randomly divided into two groups (solvent control group and amentoflavone-treated group) and subcutaneously injected with DMSO (solvent control) or amentoflavone (50 mg/kg). For survival assays and bacterial burden assays, the number of animals (n) was 10 in each group, and n = 5 for histopathology determination assays in each group. All mice were treated with three doses at 8-h intervals. Survival rates were calculated until 24 h after infection. In other assays, mice were euthanized, and infected thighs were harvested for histopathology with HE staining or homogenized for bacterial load assays at 24 h after infection.

### Antibacterial Activity Assays

Minimum inhibitory concentrations (MICs) of amentoflavone against *C. perfringens* were determined by a reference agar dilution method according to Clinical and Laboratory Standards Institute (CLSI, 2012). In growth curve assays, different concentrations of amentoflavone were supplied to early-logarithmic-phase *C. perfringens* cultures (OD600 nm = 0.3) to obtain final concentrations at 0 to 16 μg/ml. The bacteria were grown in an anaerobic environment. The absorbance of cultures were measured at 600 nm to investigate the effect of amentoflavone on growth of *C. perfringens*.

### Perfringolysin O Oligomerization Assays

Purified PFO (6 μg) were incubated with amentoflavone at final concentrations of 8 or 16 μg/ml at 37°C for 20 min in 427 μl PBS (without KCl and KH2PO4) buffer. Then, 70 μl KCl (1 mg/ml) were added, incubated at 37°C for 10 min. Three-microliters of rabbit red blood cells were added to the system and followed another incubation at 37°C for 2 min. Samples were supplied with sodium dodecyl sulfate polyacrylamide gel electrophoresis (SDS-PAGE) loading buffer (β-mercaptoethanol free) and heated at 55°C for 5 min. Western blot assays were performed as described previously (Zhao et al., 2017). A rabbit polyclonal to PFO (1:4,000 dilution, Abcam, Cambridge, USA) were used as primary antibody and a goat anti-rabbit (1:2,000 dilution) were used as secondary antibody.

### Statistical Analysis

Data are presented as the mean ± SD from three independent experiments and were further analyzed using GraphPad Prism 5.0 (GraphPad Software, CA, USA). A one-way ANOVA with the *post hoc* Tukey-Kramer multiple-comparison test was used to identify significant differences among groups (groups > 2), and an independent *t*-test was employed to identify significant differences between groups (groups = 2). Survival curves were estimated by the log-rank (Mantel-Cox) Test to assess the significance of differences in survival rates among the two groups. p < 0.05 (*) and p < 0.01 (**) were considered statistically significant.

## Results

### Amentoflavone Inhibits Hemolysis Mediated by Alpha-Toxin and Perfringolysin O

Studies have shown that both CPA and PFO can bind to red blood cells to induce hemolytic activity (Flores-Diaz and Alape-Giron, 2003; O’Brien and Melville, 2004). Here, we determined the inhibitory effects of amentoflavone on the hemolytic activity of CPA and PFO. As shown in **Figures 1B, C**, amentoflavone at concentrations of 1 and 2 μg/ml significantly decreased the hemolysis rate of CPA and PFO, respectively, indicating that the biological activities of CPA and PFO were blocked by amentoflavone. The IC50 value was 1.37 μg/ml to CPA and 2.50 μg/ml to PFO. Taken together, these results indicated that amentoflavone is an effective inhibitor against both CPA and PFO.

### Amentoflavone Protects Caco-2 Cells From Toxin-Mediated Cell Injury

**Figure 2:**
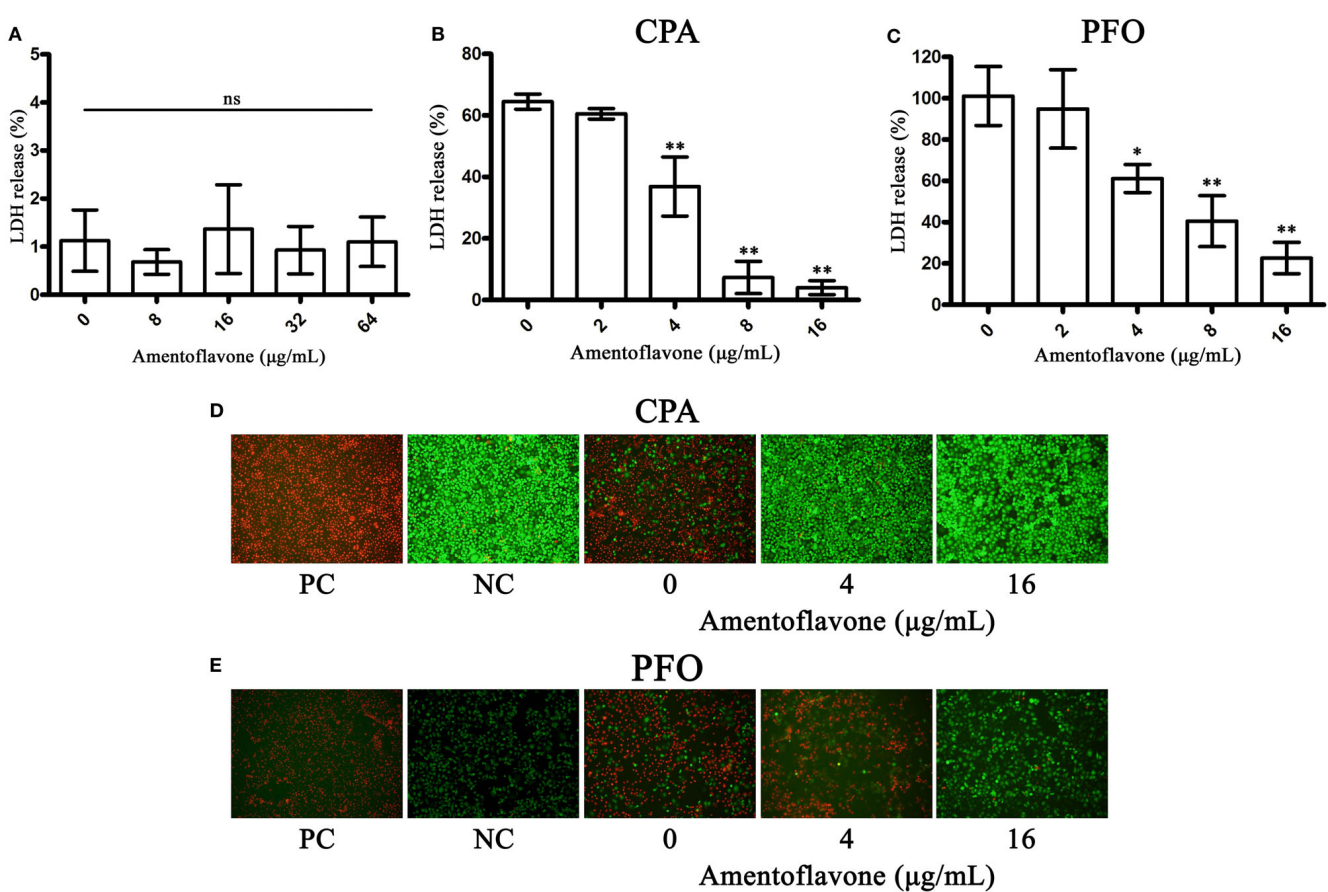
Amentoflavone protects Caco-2 cells from toxin-mediated cell injury. **(A)** LDH release by amentoflavone-treated Caco-2 cells. ns, no significant difference. **(B)** Lactate dehydrogenase (LDH) release by alpha-toxin (CPA)-treated Caco-2 cells in the presence of amentoflavone. **(C)** LDH release by perfringolysin O (PFO)-treated Caco-2 cells in the presence of amentoflavone. LDH release was measured to evaluate toxin-mediated cytotoxicity in the presence of amentoflavone in each group. Data are shown as the mean ± SD from three independent experiments. * indicates P < 0.05 and ** indicates P < 0.01 compared to the drug-free group. **(D)** LIVE/DEAD-stained Caco-2 cells after incubation with CPA following amentoflavone treatment. **(E)** LIVE/DEAD-stained Caco-2 cells after incubation with PFO following amentoflavone treatment. Live cells showed green fluorescence, and dead cells showed red fluorescence. PC, positive control group; NC, negative control group.

CPA and PFO are responsible for cytotoxicity toward mammal cells (Flores-Diaz and Alape-Giron, 2003; Verherstraeten et al., 2013). In hemolytic activity assays, we found that amentoflavone could inhibit the hemolytic activity of CPA and PFO; thus, we further explored the protective effects of amentoflavone on toxin-mediated cytotoxicity toward Caco-2 cells. Firstly, the cytotoxicity of amentoflavone on Caco-2 cells was determined. As shown in **Figure 2A**, amentoflavone (ranging from 0 to 64 μg/ml) had no cytotoxicity on Caco-2 cells. Consistent with our expectations, amentoflavone significantly protected cells from cytotoxicity mediated by CPA (**Figures 2B, D**) and PFO (**Figures 2C, E**) at a concentration of 4 μg/ml. In LDH release assays, only 4.03 and 22.63% of cells were dead in the coculture system of Caco-2 cells with CPA and PFO, respectively, when incubated with 4 μg/ml amentoflavone. The IC50 value was 2.18 μg/ml to CPA and 6.35 μg/ml to PFO. In LIVE/DEAD assays, most cells were strained with green fluorescence, indicating that live cells accounted for the majority. Taken together, our results suggest that amentoflavone treatment protects Caco-2 cells from injuries mediated by CPA or PFO.

### Protective Effect of Amentoflavone Against *Clostridium perfringens* infection

**Figure 3:**
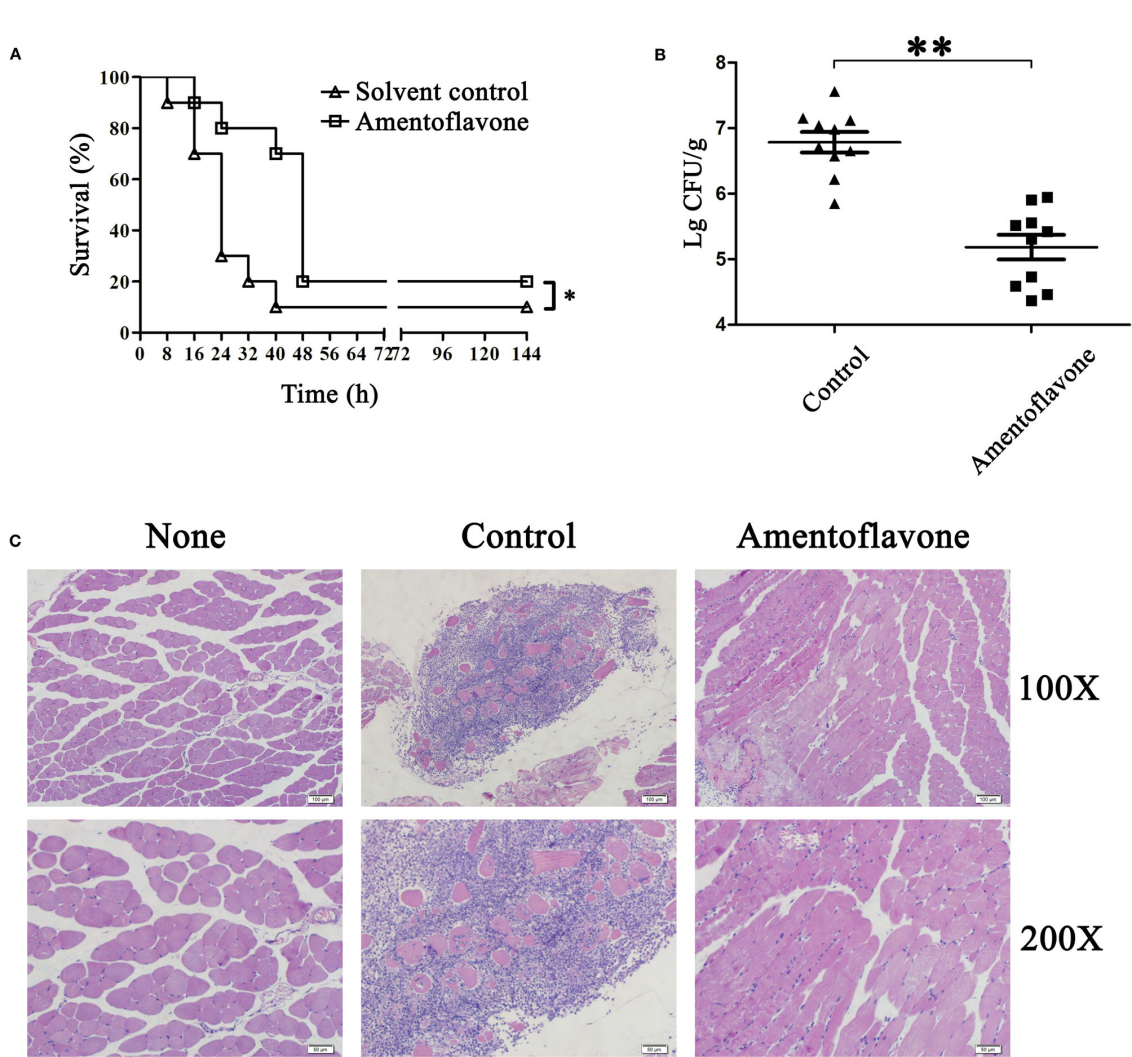
Protective effect of amentoflavone against *Clostridium perfringens* infection. **(A)** Survival curves of *C. perfringens*-infected mice with or without amentoflavone treatment. Mice were intramuscularly injected in the thigh muscle with *C. perfringens* ATCC13124 and then subcutaneously injected with dimethyl sulfoxide (DMSO) (solvent control) or amentoflavone (50 mg/kg) (n = 10). The survival rate was plotted with the Kaplan-Meier method, and the generalized Wilcoxon test was used to assess the significance of differences in survival rates among the two groups. * indicates P < 0.05 compared to the control group. **(B)** Bacterial survival of *C. perfringens* in infected tissues between the two groups. Dashes represent the mean value of the number of surviving bacteria (n = 10). ** indicates P < 0.01 compared to the control group. **(C)** Histopathology changes in *C. perfringens-*infected tissues in mice.

To evaluate the *in vivo* effect of amentoflavone in the development of gas gangrene, mice were intramuscularly infected with *C. perfringens* and subcutaneously injected with amentoflavone. There was a statistically significant difference between the two groups after three doses of amentoflavone treatment for survival, bacterial burden, and histopathological analysis. Mice in the amentoflavone treatment group had a survival rate of 80% at 24 h, compared with 30% in the solvent group (**Figure 3A**). The peak of the death period was 48 h after amentoflavone treatment compared with 24 h in the solvent group, which indicating three doses of amentoflavone treatment could extend the survival time for 24 h. In bacterial burden assays, the number of bacteria colonized in tissues of the amentoflavone treatment group were significantly lower than those in the control group (5.18 ± 0.58 Lg CFU/g *vs*. 6.79 ± 0.48 Lg CFU/g), which demonstrated that the survival of bacteria in host tissue was decreased by amentoflavone (**Figure 3B**).

In the histopathology assays, mice of the control group infected with *C. perfringens* ATCC13124 expressed typical pathological damage, with numerous leukocytes infiltrating these tissues (Awad et al., 2001). After administration of amentoflavone, little pathological damage was apparent in a given visible field (**Figure 3C**). All these results clearly demonstrated that amentoflavone provided significant therapeutic protection against gas gangrene caused by *C. perfringens* infection.

### Amentoflavone Exhibits No Antibacterial Activity Against *Clostridium perfringens*

**Figure 4:**
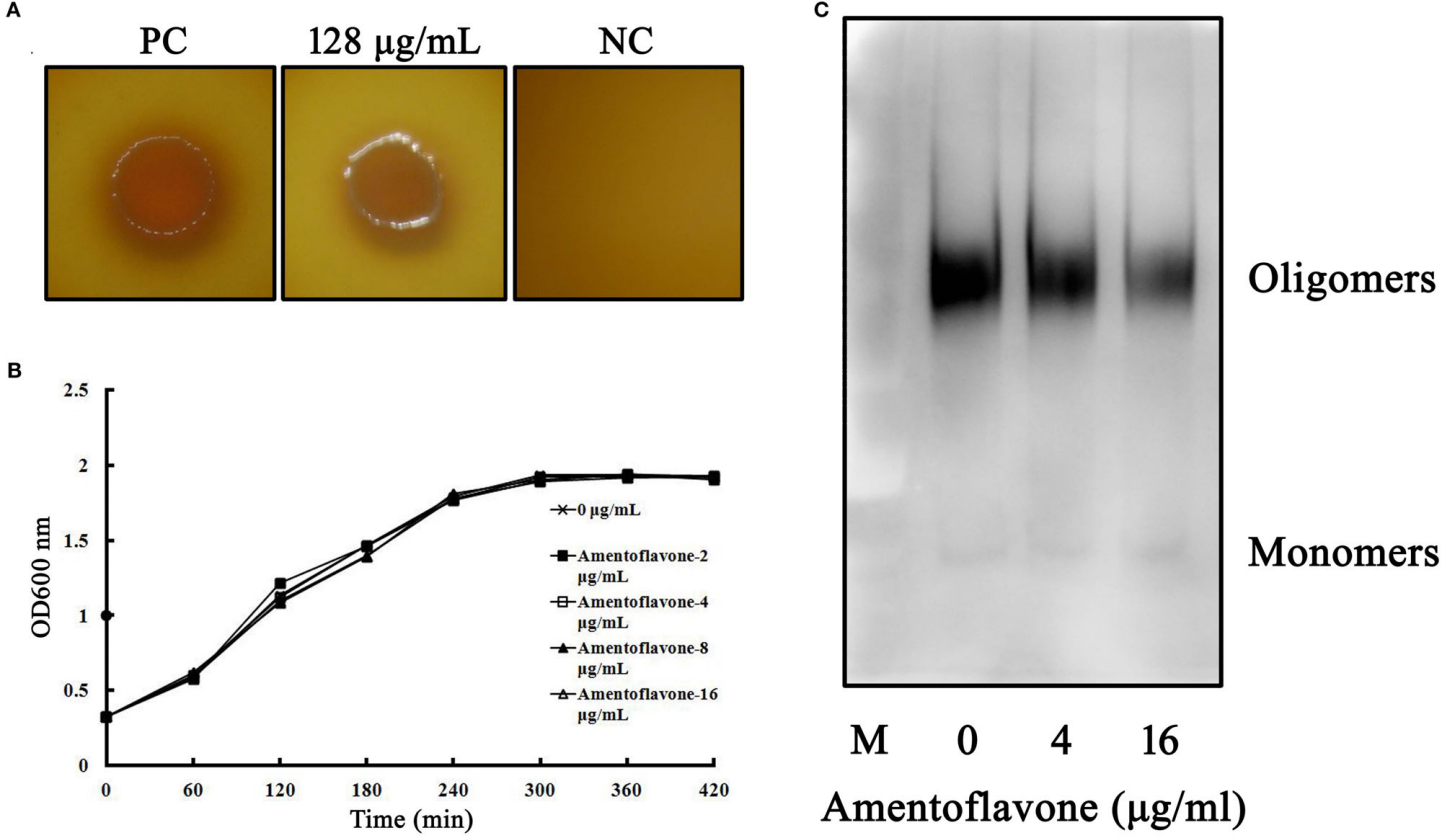
**(A)** Minimum inhibitory concentration (MIC) assays of amentoflavone against tested *Clostridium perfringens* strain. The colony formation on agar plates was used to assess the MIC values. Abbreviations: PC, positive control group, added with bacteria but without amentoflavone; NC, negative control group, no amentoflavone and bacteria was added. **(B)** Growth curves of *C. perfringens* treated with amentoflavone. *C. perfringens* were treated with different concentrations of amentoflavone (0 to 16 μg/ml). The absorbance of cultures was measured at 600 nm to investigate the effect of amentoflavone on growth of *C. perfringens*. **(C)** Amentoflavone weakens perfringolysin O (PFO) oligomerization. The formation of PFO oligomers was explored using Western blot assays following amentoflavone treatment.

In MIC assays, significant colony formations were observed in the positive group and the amentoflavone-treated group (128 μg/ml), while no observable bacterial growth was viewed in the negative control group, demonstrating that the MIC value of amentoflavone against *C. perfringens* was greater than 128 μg/ml (**Figure 4A**). In the growth curves assays, no significant differences were observed in the growth of *C. perfringens* with or without amentoflavone. Thus, amentoflavone (at a concentration below 16 μg/ml) had no effect on the growth of the tested *C. perfringens* (**Figure 4B**).

### Amentoflavone Weakens Perfringolysin O Oligomerization

In hemolytic activity and cytotoxicity assays above, we found that amentoflavone could inhibited the biological activities of PFO. To further investigate the mechanism of amentoflavone on PFO activities, we determined the effects of amentoflavone on PFO oligomerization. Our result showed that the oligomerization bands were reduced when the concentrations of amentoflavone increased, suggesting that amentoflavone interfered with the progress of PFO oligomerization and hindered the formation of pore in the cell membrane to lyse cells (**Figure 4C**).

## Discussion

Gas gangrene is a fatal toxin-mediated disease with a very rapid progression (usually causing death within 24 to 48 h after infection) (Stevens, 2000). CPA and PFO are essential virulence factors in gas gangrene pathology. Studies have demonstrated the role of CPA and PFO in the development of gas gangrene and the synergistic effects of these two toxins in causing tissue damage and triggering disease (Awad et al., 2001; Verherstraeten et al., 2013). The critical role of CPA or PFO in the disease of gas gangrene was previously studied (Stevens et al., 1997; Awad et al., 2001; Flores-Diaz and Alape-Giron, 2003; O’Brien and Melville, 2004). Here, we found that amentoflavone could neutralize the hemolytic effects mediated by CPA or PFO and the neutralization effect mediates effective protection on target cells *in vitro*. Previous studies have reported that CPA and PFO are pore-forming toxins which can bind to membranes and oligomerize into a pore complex to lyse cells (Stevens and Bryant, 2002; Navarro et al., 2018). Hindered the oligomerization of pore complex in the cell membrane may remove the pore-forming activity of these two toxins. Thus, the inhibition of PFO oligomerization may be the mechanism of amentoflavone on PFO neutralization. However, the mechanism of amentoflavone affecting CPA or PFO activity and how amentoflavone interacts with these two toxins are still unclear.

To date, there have been numerous studies on the development and use of natural compounds against bacterial infection *via* targeting toxins and other virulence factors (Qiu et al., 2012; Zhang et al., 2014; Wang et al., 2015). Zhao et al. reported the anticytotoxin effects of amentoflavone on pneumolysin, a devastating bacterial protein toxin of *Streptococcus pneumoniae* and a member of the CDC family (Alouf, 2000; Zhao et al., 2017). Besides, study of Shen et al. showed that amentoflavone effectively inhibited suilysin-mediated hemolysis, a member of the CDC family toxin from *Streptococcus suis* (Shen et al., 2018). The finding that amentoflavone targets PFO activity was expected since PFO is also a member of the CDC family. Here, our data support the inhibitory effect of amentoflavone on CDC family toxins (Zhao et al., 2017; Shen et al., 2018).

Previous studies have reported that CPA and PFO are required for *C. perfringens*-induced gas gangrene and bacterial survival in host tissues. A *cpa* and *pfoA* double mutant was unable to induce most of the histopathological features typical of gas gangrene (Awad et al., 2001). In this report, mice in the control group died rapidly, and only 30% of mice survived after 24 h of infection. In contrast, mice in the amentoflavone treatment group had a significantly greater survival rate of 80%, indicating that amentoflavone could delay the peak of the death period (24 h in the control group to 48 h in the amentoflavone treated group) and extend the precious time available for clinical rescue. In addition, amentoflavone treatment could decrease the survival of bacteria in infected tissues and alleviate histological damage. Taken together, these results demonstrate that the application of amentoflavone in infected mice showed increased animal survival and decreased bacterial burden in tissues, indicating that antivirulence activity is effective for protection *in vivo*.

In conclusion, the results of the study demonstrated that amentoflavone could block the hemolysis and cytotoxicity induced by CPA and PFO *in vitro*, thereby mediating significant protection in a mouse gas gangrene model, efficient bacterial clearance in tissues, and alleviation of histological damage *in vivo*. Our data showed that amentoflavone may have considerable therapeutic value for the prevention and management of *C. perfringens* infection by reducing CPA and PFO-mediated virulence.

## Data Availability Statement

The datasets generated for this study are available on request to the corresponding authors.

## Ethics Statement

The animal study was reviewed and approved by Jilin University Institutional Animal Care Committee.

## Author Contributions

JW, XB, XD and SL designed the study and wrote the manuscript; SL, XY, HZ, JZ, YZ, TW and NH performed the experiments; JW, XB and XY analyzed the data.

## Funding

This work was supported by the National Key Research and Development Program of China (2018YFD0500300) and the National Natural Science Foundation of China (31772782 and 81861138046).

## Conflict of Interest

The authors declare that the research was conducted in the absence of any commercial or financial relationships that could be construed as a potential conflict of interest.
